# Annihilating Pores in the Desired Layer of a Porous Silicon Bilayer with Different Porosities for Layer Transfer

**DOI:** 10.1038/s41598-019-49119-8

**Published:** 2019-09-02

**Authors:** C.-C. Chiang, Benjamin T.-H. Lee

**Affiliations:** Department of Mechanical Engineering, National Central University, Taoyuan City, Taiwan

**Keywords:** Porous materials, Synthesis and processing

## Abstract

A silicon layer that is tens of micrometers thick on a handle substrate is desired for applications involving power devices, microelectromechanical systems (MEMS), highly efficient silicon solar cells (<50 µm), etc. In general, if the initial silicon layer obtained from the layer transfer process using the etch-stop or ion-cut techniques, which may provide very accurate thickness control, is too thin, then additional epitaxial growth is required to increase the thickness of the silicon layer. However, epitaxial growth under strict predeposition conditions is a time-consuming and expensive process. On the other hand, producing porous silicon via anodization in a hydrofluoric acid solution offers an efficient way to control the dimensions of the generated pores directly on the nano- or macroscale via the current density. When sintering the porous layer via high-temperature argon annealing, the porosity of the porous layer determines whether this porous layer can serve as a device layer or a separation layer. In addition, it is clearly easier to create a transferred layer ten of micrometers thick via anodization than by ion implantation and/or epitaxial deposition.

## Introduction

The layer transfer technique, based on a defined cutting position and wafer-bonding technology^1,2^, can transfer a layer from a crystalline substrate onto a desired substrate. This technique has great flexibility in terms of a sub-microscale thickness—0.5 μm, for example—but maintains the crystalline quality and properties of the original substrate, which has proven helpful in the development of some innovative applications such as hybrid integration of IC and MEMS^3^, high-efficiency solar cells^4,5^, and device layer transfer^6–8^.

One of the best examples of the application of the layer transfer technique is in the fabrication of SOI (silicon on insulator) materials. The available manufacturing methods are considerably different^1,2,9–11^ for making different SOI thicknesses for various applications. For example, to fabricate ultrathin SOI materials (silicon thickness <0.1 μm), ion-cut technology is a quite mature and appropriate technology. For the fabrication of thick SOI materials (10 μm <SOI silicon thickness <100 μm), fine grinding and polishing is most frequently used. When the SOI silicon thickness is between 1 μm and 10 μm, the manufacturing is more difficult than for other thicknesses. Typically, the procedure combines ion-cut processing with an epitaxial growth technology for fabrication because such a thickness range is outside the regular scope of polishing-thinning or hydrogen ion-cutting. However, the cost of this type of thick SOI manufacturing may be increased greatly if the layer transfer process involves ion implantation and epitaxial growth.

The reason for developing a layer-transfer process for thin-film solar cells is that a thinner wafer thickness of the silicon solar cells corresponds to a greater reduction in the consumption of the silicon substrate and a greater increase in cell efficiencies^12^. These benefits have prompted many renewable energy research teams to study kerfless techniques^13,14^ for producing silicon layers with thicknesses of tens of micrometers (<50 μm) with layer-transfer processing. When manufacturing solar cells, the two processes of ion-cut and epitaxial growth are ideally used as little as possible. Here, we demonstrate a layer-transfer method using only an electrochemical process to produce a porous silicon bilayer or multilayer as both the device layer and the separation layer while omitting the ion implantation and epitaxial growth steps.

Since 1995, the ELTRAN process developed by Sato *et al*.^15^ has utilized a porous silicon layer as a separation layer to detach a predeposited silicon layer to transfer this layer onto a desired substrate for many applications^16–18^. When applying this process to the manufacture of thin-film solar cells, the silicon layer needs to grow into a layer tens of micrometers thick via the silicon epitaxial growth technique. Based on the concept of interaction between the porous silicon separation layer and silicon epitaxial growth, various extensive methods have been developed to grow the top device layer on the separation layer, such as SONY’s sintered porous silicon (SPS) method^19^, kerfless layer transfer^13^, and quasi-monocrystalline porous silicon (QMPS)^20,21^. In addition, IMAC uses thermal stresses to delaminate the thick silicon layer from a silicon ingot predeposited by a thick metal layer with a large difference in the thermal expansion coefficient (e.g., silver)^22,23^. A multilayer porous silicon structure incorporating recrystallization techniques is used in an alternative manufacturing method.

There are several approaches to fabricating a multilayer porous silicon structure^24^, such as performing anodization with alternating doping concentration layers, anodization with alternating operation temperatures, or anodization with alternating applied current densities. Because the dissolution of silicon preferentially occurs at the pore tips, where the field is concentrated^25,26^, the porosity of the layer formed previously is almost unchanged during subsequent etching. To create such multilayer structures, a variable current density is applied.

This layer transfer method prepares a transferable silicon layer in three main steps. First, it produces a low- to high-porosity porous silicon bilayer by applying variable current densities to change the porosity during anodization processing. We define the thickness of the device layer via time control. The optimum thickness of the top porous silicon layer used in this technique is in the range of 1 to 50 μm. Second, it applies a high-temperature argon annealing technique^27,28^ to close the pores in the top porous silicon layer, thereby converting it into a defect-free crystalline layer. Finally, it transfers the fused top silicon layer of the device onto the desired substrate using the wafer-bonding technique.

The principle of using varying currents to create different-porosity layers is based on the Lehmann-Gösele model^26,29^. They suggested that in an acidic electrolyte containing fluoride ions, the application of an anodic potential can cause an electrochemical dissolution reaction (i.e., one involving SiF_6_^2−^) on the surface of the silicon substrate. The surface of the silicon substrate then forms porous silicon and Si-F bonds. The chemical reaction equations are as follows:

1.1 $${\rm{Si}}+2{\rm{HF}}+{{\rm{h}}}^{+}\to {{\rm{SiF}}}_{2}+{{\rm{H}}}_{2}+{{\rm{e}}}^{-}$$

1.2 $${{\rm{SiF}}}_{2}+2{\rm{HF}}\to {{\rm{SiF}}}_{4}+{{\rm{H}}}_{2}$$

1.3 $${{\rm{SiF}}}_{4}+2{\rm{HF}}\to {{{\rm{SiF}}}_{6}}^{2-}+{{\rm{2H}}}^{+}$$

When the applied current varies, changes in the rate of the dissolution reaction change the porosity.

After the porous silicon bilayer forms, argon annealing may be used to convert the top porous silicon layer into a defect-free crystalline layer through reorganizing and annihilating pores. Milenkovic *et al*.^30^ reported that based on an X-ray diffraction rocking curve, a 950 °C annealing treatment can shift the peak of as-etched porous silicon to higher diffraction angles, changing a tensile out-of-plane strain into a compressive out-of-plane strain. Because tensile strain usually increases with increasing porosity and mean pore radius, this finding indicates that the pores become much smaller if the strain is changed from tensile to compressive. Moreover, at a higher temperature of 1150 °C, the porous layer may “relax” to greatly reduce the compressive strain. The conversion efficiency of thin-film solar cells fabricated by the shallow emitter formation process^31^ is 12.5% when using the silicon substrate prepared by this layer-transfer technique. However, the conversion efficiency may be higher if not limited by the performance of the fabricating equipment.

## Experiment and Theory

**Figure 1:**
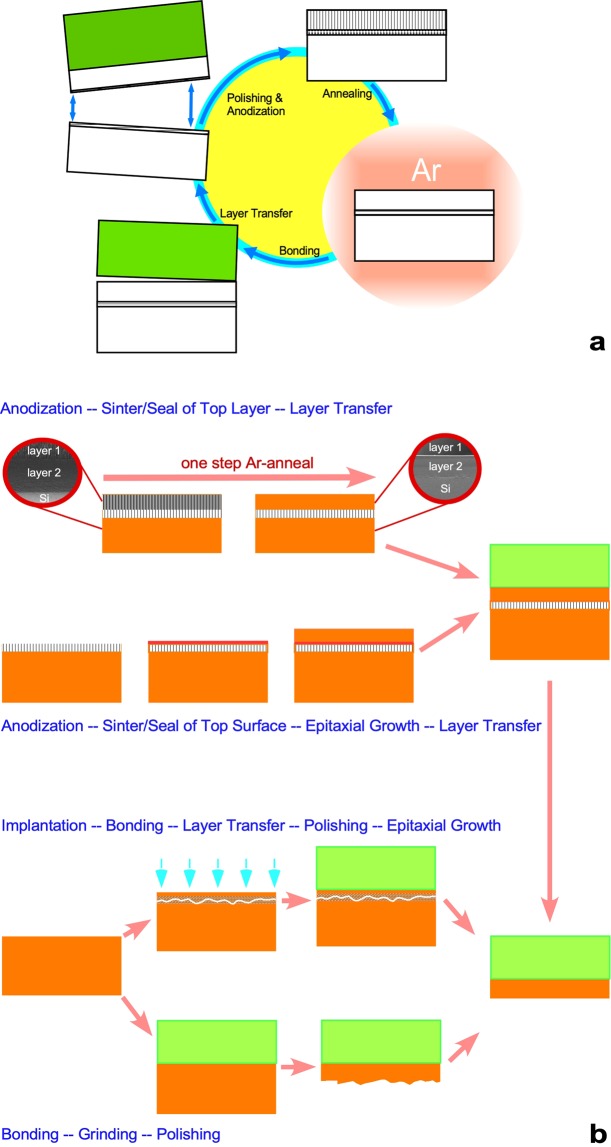
The flowchart for a thick silicon layer transfer technique enabled merely by anodizing the silicon substrate and a comparison with other layer-transfer-based processes. (**a**) The steps in forming a device layer on a host substrate during layer transfer are (1) forming the first porous silicon layer with a low porosity, (2) forming the second porous silicon layer with a high porosity, (3) annealing the anodized specimen in argon at a high temperature to convert the second porous silicon layer into a device layer, (4) performing wafer bonding to combine the specimen with a handle substrate, and (5) splitting the device layer from the host substrate via the separation layer. The surface of the remaining host substrate may be polished and recycled as a new host substrate. (**b**) The thick layer-transfer technology in the study (top), with a comparison to other layer transfer techniques (anodization + epitaxial growth (second), ion-cut process + epitaxial growth (third), and grinding/polishing (last)). The thick layer-transfer technology shown at the top omits the epitaxial growth processing and recovers the host substrate.

In Fig. 1, we illustrate the steps of the layer transfer process we developed and make a comparison with other layer-transfer technologies. The sequence of the layer-transfer process based on a porous silicon-based structure is depicted schematically in Fig. 1a. First, the use of an HF-based anodization process forms a porous silicon stack with different porosities on the surface of the silicon specimen by adjusting the current density. The demonstrated porous silicon stack is in a bilayer structure created by a nearly 50 μm thick low-porosity device layer and a 10–15 μm thick high-porosity separation layer. After anodization, the specimen is blown dry by argon in a quartz tube and then annealed in argon at an ambient temperature of 950–1100 °C, which results in sintering of the porous silicon. Sintering the porous silicon not only seals the pores to form a crystalline layer in the low-porosity layer but also causes the Ostwald ripening process to occur, forming a split plane in the high-porosity layer. Figure 1b displays a comparison with other layer-transfer technologies, such as the similar process of first forming porous silicon as a separation layer and epitaxially growing a thick silicon layer as a device layer. The layer-transfer technology integrated with anodization, argon annealing, and wafer bonding has at least two benefits—omitting expensive epitaxial growth processing and recycling the host substrate.

In the study, all of the specimens were cut into squares of 5.0 × 5.0 cm^2^ from a prime grade, boron-doped, 1~10 Ω – cm (100) silicon wafer. The radius of the anodized region was a disk with a radius of 13.6 mm (area = 5.81 mm^2^). The specimens were cleaned in RCA (NH_4_OH:H_2_O_2_:H_2_O = 1:1:5) and then anodized in a Teflon electrolytic tank filled with hydrofluoride solution (HF at 42%:C_2_H_5_OH at 99.5% = 1:1).

The use of an applied current (100–300 mA; i.e., current density = 17–52 mA/cm^2^) controlled the porosity of the porous layer, and the anodization time (10–60 minutes) determined the thickness of the porous layer. In this study, the silicon specimen was immersed in an HF-based electrolyte solution. Then, an initial anodization step was carried out under the first condition of 100–150 mA, and after 20–50 minutes, anodization was conducted at 300 mA for 10–20 minutes. The initial applied current was lower than the subsequent applied current to create a low- to high-porosity bilayer structure in the porous layer.

The method we used to measure the porosity (*P*) of the porous silicon layer was based on the weight measurement of the mass of the specimen before (original mass, *m*_0_) and after (*m*_1_) anodization and the mass (*m*_2_) after merely removing the porous layer. The porosity can be obtained by the equation:

$$p=\frac{{m}_{0}-{m}_{1}}{{m}_{0}-{m}_{2}}$$

Inspection and measurement with TEM, SEM, XRD and polar plots were performed to check the material quality of the transferred layer, and the results are explicated in the associated figure descriptions.

## Results and Discussion

**Figure 2:**
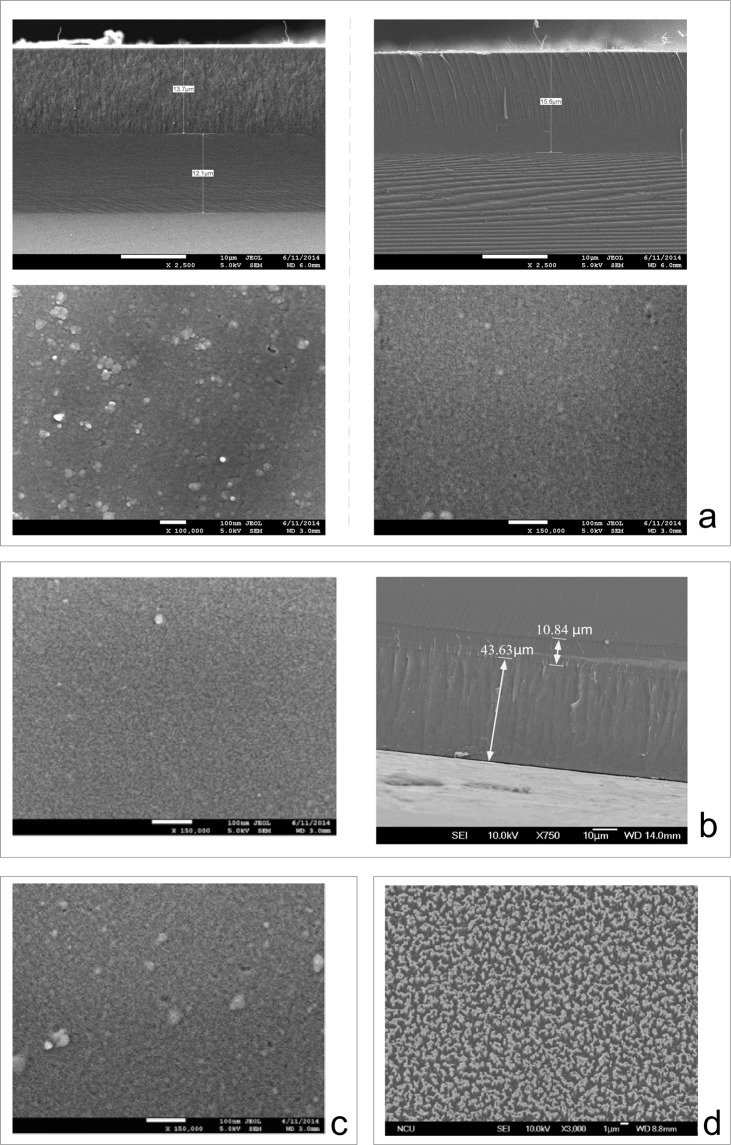
SEM observation of porous silicon bilayers with different porosities obtained using different current densities in an anodization sequence. (**a**) SEM observation of the side view (upper row, right and left) and top view (lower row, right and left) of the porous silicon bilayer formed by two current densities. SEM images of the side view of the 1–10 ohm-cm, p-type silicon specimens obtained by anodization at 8.6 mA/cm^2^ for 10 minutes and then at 25.8 mA/cm^2^ for 20 minutes. After anodization, the specimens were (before, left; after, right) annealed in argon at 1000°C for one hour. After annealing in argon, the porous column was sintered into a solid layer, which caused the scratched appearance during the cutting portion of the SEM sample preparation. The morphology is different from that in which the porous column exhibited a ruptured powdery trace before annealing, as shown in the figures in the upper row (the right panel and the underlayer in the left panel). (Scale bar = 10 microns). (**b**) The top view (left) and the side view (right) of the bilayer formation of porous silicon using SEM: No pores appeared on the top surface of the upper porous layer. The upper silicon porous layer is 43.63 μm thick, and the bottom porous silicon layer is 10.84 μm thick after anodizing a 1–10 ohm-cm p-type silicon specimen at 17.2 mA/cm^2^ of current density for 50 minutes and then at 52 mA/cm^2^ of current density for 10 minutes. (Scale bar = 100 nm). (**c**) The top view of the porous silicon bilayer obtained using SEM. The silicon porous bilayer was formed by anodizing a 1–10 ohm-cm p-type silicon specimen at 8.6 mA/cm^2^ of current density for 10 minutes and then at 25.8 mA/cm^2^ of current density for 20 minutes (scale bar = 100 nm). There are some small pores (approximately 10 nm in diameter) on the top surface of the upper porous layer. (**d**) The SEM top view of an as-split surface on the transferred layer after annealing. The separation layer was formed from the last porous silicon layer with a porosity of 56.54%. There are many fractured silicon pillars remaining on the 3–5 μm surface (scale bar = 1.0 micron).

After anodization, the initial 43.6 μm porous layer had a porosity of 20–30% (50 minutes, 17 mA/cm^2^), and the later-forming 22 μm porous layer had a doubled porosity of 50–60% (10 minutes, 52 mA/cm^2^). The last porous silicon layer serves as a separation layer between the device layer formed from the initial porous layer and the silicon substrate. The reduction in the thickness of the separation layer was approximately 30–40% after annealing, so that the final thickness was close to 14 μm. The 950–1100 °C argon annealing process was used to fill the upper porous layer by enabling the in-diffusion of neighboring silicon atoms and promoting the Ostwald ripening of pores in the bottom porous layers. As shown in the scanning electron microscope (SEM) pictures in Fig. 2, the highly porous buried layer yielded large cavities and, thus, a mechanically weak layer, but the low-porosity layer was sealed, resulting in a crystalline device layer. In the appendix, we show that on a silicon wafer anodized under different current densities, different void-ratios are presented. After high-temperature annealing of the anodized specimens in argon, the layer with a lower porosity was sintered to a bulk layer with recrystallization, while the layer with a high porosity exhibited a void-filled status. We choose the best mode to illustrate the layer transfer processing utilizing the sintering characteristics of bilayer porous silicon, as shown in Fig. 2.

In Fig. 2a, the upper silicon porous layer is 13.7 μm thick, and the bottom porous silicon layer is 12.1 μm thick. They were created by anodizing the silicon specimen at 8.6 mA/cm^2^ for 10 minutes and then at 25.8 mA/cm^2^ for 20 minutes. The anodized specimens were then annealed in argon at 1000°C for one hour. The SEM images show the different surface morphologies caused by SEM sample preparation between the unsintered porous layer (ruptured trace) and the sintered porous layer (scratch track). Adjusting the final current density can modify the porosity of the last porous silicon layer. However, we found several pores on the surface of the top porous silicon layer (device layer), as shown in Fig. 2c. These pores disappeared at the highest and final current density of 52 mA/cm^2^, as shown in Fig. 2b (left). It is well known that larger currents may more easily pass through the top porous silicon layer without reacting with the pore wall, meaning that the bottom silicon substrate will be anodized to form a new porous layer with a larger porosity^24,32^.

When the anodization time of 17.2 mA/cm^2^ was prolonged to 50 minutes, the thickness of the top porous silicon layer after annealing was 43.6 μm, which is very close to the target thickness of 50 μm, as shown in Fig. 2 (right). The follow-up anodization time can be fine-tuned via testing to ensure that the thickness of the porous silicon layer after annealing is as close as possible to 50 μm.

In the oxygen-plasma-activated wafer bonding technique, the Ar-annealed silicon specimen was bonded to a handle silicon substrate covered with an oxide layer by annealing at 200 °C for 5 hours. As shown in the SEM image in Fig. 2d, which shows the top view of the as-split surface on the transferred device layer, there are many fractured silicon pillars remaining on the surface.

**Figure 3:**
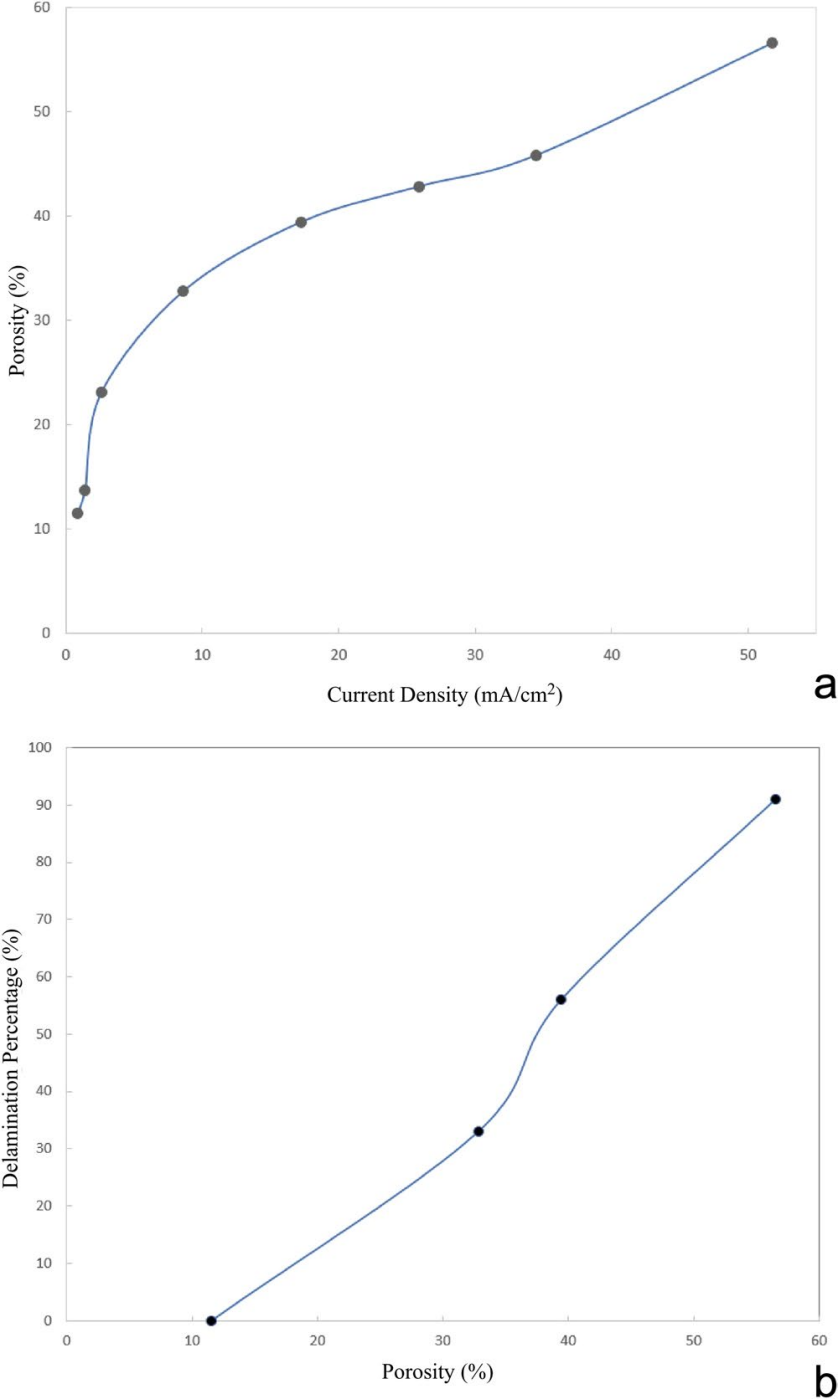
The porosity and growth rate of porous silicon as a function of current density in a p-type silicon substrate anodized in the HF/C_2_H_5_OH electrolyte. (**b**) Porous silicon layers with various porosities formed after anodizing p-type Si for 15 minutes. The suitable range for producing a porous silicon layer with a low porosity is below 100 mA (=17 mA/cm^2^) and that for producing a porous silicon layer with a high porosity is 200–300 mA (=34–52 mA/cm^2^). (**b**) Porosity is the important influencing factor for the delamination percentage after bonding the surface of the device layer to a handle substrate.

Figure 3a displays a typical graph of the porosity as a function of the current density for the process of anodizing p-type Si for 15 minutes. The appropriate current density range for forming a low-porosity layer^33^ as the device layer is 5.2–26 mA/cm^2^ and the range for forming a high-porosity layer proposed to serve as the separation layer is 34–52 mA/cm^2^. The peeling percentage is strongly affected by the porosity of the separation layer, as shown in Fig. 3b. The higher the porosity, the more and larger the pores will be, and the lower the strength of the material will be, resulting in easier delamination.

The argon high temperature annealing^34–36^ in this study has the following functions: (a) sealing and fusing the pores in the device layer and (b) automatically smoothing the sealed surface by driving out the hydrogen atoms via breaking the Si-H bonds attached to the surfaces of the pore walls. The passivation of the hydrogen molecule is believed to be the obstacle in eliminating the pores completely during anodization. Argon has a strong effect on splitting off the silicon-hydrogen bonds to promote the recovery of silicon crystallization. A structural model proposed by T. W. Sigmon’s research group^37^ for removing the amorphous-crystalline interface suggests that cooperative interfacial bond breaking and rearrangement may promote the regrowth process of the crystalline layer. It has been found that high-temperature argon annealing is able to eliminate crystal-originated particles (COPs) within one hundredth of a micrometer of the surface of a Czochralski (CZ)-grown silicon wafer. Furthermore, in the case of a silicon layer formed by a chemical vapor deposition (CVD) process mixed with argon gas, as disclosed in U.S. patent no. 9,287,137, argon is able to drive out dangling silicon-silicon bonds or silicon-hydrogen bonds so that strong silicon-silicon bonds form a hydrogen-free silicon layer. On the other hand, by annealing at a high temperature for a sufficiently long time, the long-range transport of silicon atoms causes silicon atoms in the neighboring regions to fill in the pores in the sponge-like structure, i.e., undergo vacancy diffusion to the surface. The dependence of the growth rate (filling rate) on the substrate orientation should adhere to essentially a geometrical argument in which the regrowth rate-determining step might be equivalent to the time required to grow a row of atoms along the densest (111) planes on the two neighboring crystal surfaces.

**Figure 4:**
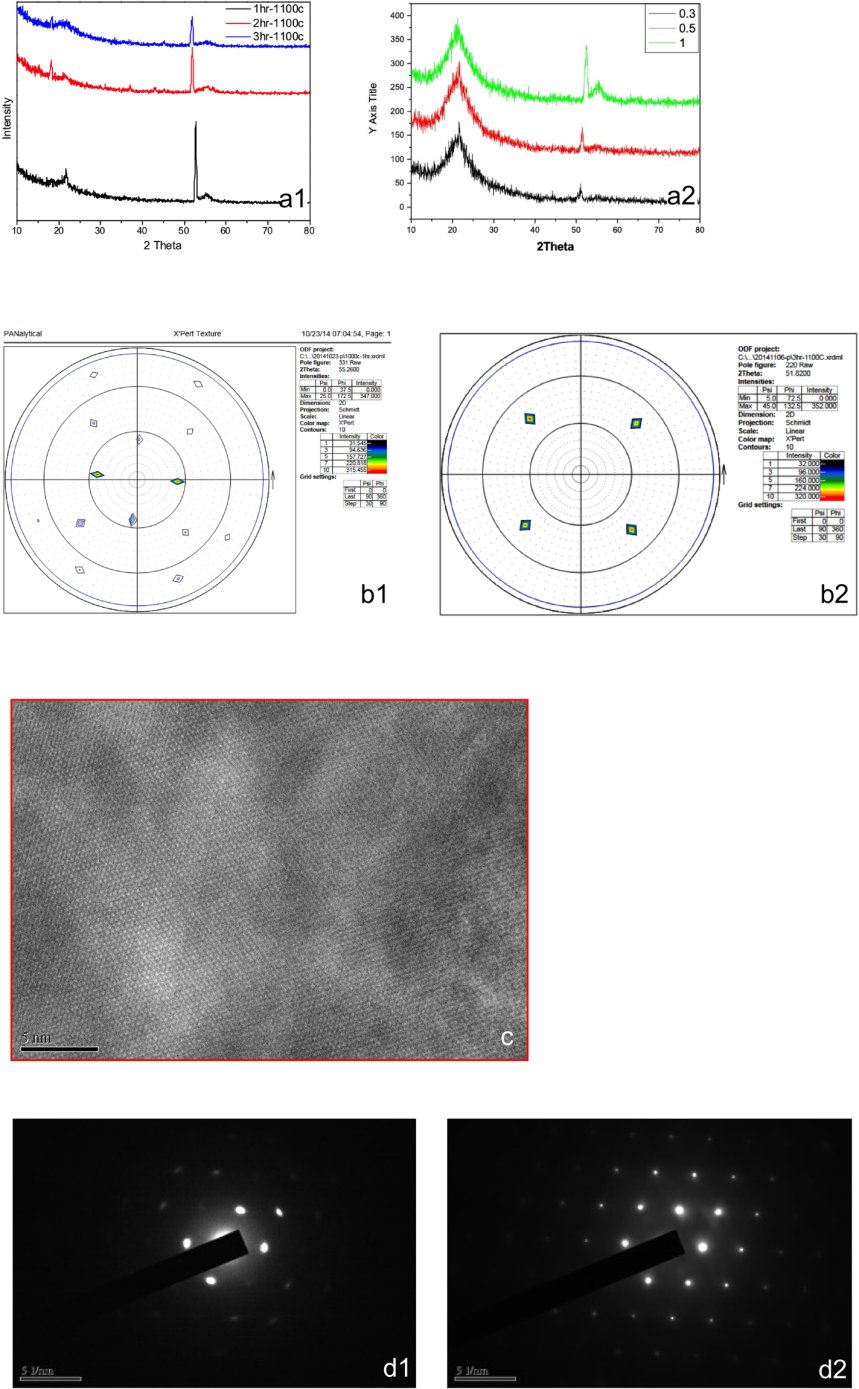
The results of X-ray diffraction (XRD), as measured by the PANalytical X′Pert Pro Multi-Purpose Diffractometer, transmission electron microscopy (TEM), and selected area (electron) diffraction (SAED) for evaluating the crystalline quality of the silicon layer annealed at 1000 °C and 1100 °C, in an argon atmosphere. (**a**) X-ray diffraction (XRD) pattern of the silicon layer after Ar annealing. The XRD pattern shows that the crystalline quality of the porous silicon layer obtained after Ar annealing at 1100 °C for 3 hours is better (A1) than the crystal quality obtained under the same conditions at 1000 °C (A2). The leftmost peak on both graphs is caused by oxidation. (**b**) PANalytical X′ Pert Pro measurements of the crystalline quality of the silicon layer obtained under various annealing conditions exhibited miscellaneous crystal plane orientations, indicating that at 1000 °C, vacancies might not be eliminated completely (B1); however, these vacancies were eliminated completely at 1100 °C for 3 hours (B2). (**c**) TEM observation displaying perfect silicon crystals after Ar annealing for 3 hours. (**d**) Selected-area (electron) diffraction of the (100) silicon device layer before (D1) and after (D2) Ar-annealing at 1100 °C for 3 hours.

As discussed above, by employing the technique of argon annealing, thermally diffused silicon-to-silicon bonding fills in and eliminates the sponge-like structure inside the top, porous silicon layer and then enables a complete recovery of crystallization. In Fig. 4a, the X-ray diffraction (XRD) pattern of the silicon layer shows that the crystalline quality of the porous silicon layer obtained after Ar annealing at 1100 °C for 3 hours is better than the crystal quality obtained at 1000 °C. The leftmost peaks in the two small figures, a1 and a2, are caused by oxidation. To construct Fig. 4b, a PANalytical X′ Pert Pro was used to examine the crystallinity of the silicon layer. In the map of PANalytical X′ Pert Pro measurements, the specimens annealed at 1000 °C had miscellaneous crystal plane orientations, indicating that vacancies might not be eliminated completely, as shown in Figure b1. However, the map shows a clear and uniform crystal plane orientation at 1100 °C annealing for 3 hours in Figure b2.

Figure 4c provides direct evidence of the high quality of the device layer after argon annealing at 1100 °C for 3 hours via transmission electron microscope observation (TEM; 4c). The selected area (electron) diffraction (SAED), as shown in Fig. 4d, reveals a single crystal structure with pores (d1) before annealing and no pores (d2) after annealing.

### Summary

In an anodizing process, the porosity and thickness of porous silicon are determined by the magnitude of the current density and the anodization time, respectively. The top porous layer with the lower porosity in the bilayer structure can be filled and reorganized to form a perfect crystalline layer via high-temperature argon annealing. This perfect layer may come about because the high-temperature argon annealing process may decompose some native silicon dioxide if it exists due to exposure to air. Argon annealing not only enhances the out-diffusion of oxygen but also intensifies Si-H bond rearrangement to remove hydrogen on the silicon surface and then promotes the in-diffusion of silicon atoms to fill and seal tiny pores. However, in the higher-porosity porous silicon layer, the Oswald ripening process reorganizes many of the pores into large cavities, causing a brittle structure that is suitable for use as a separation layer for layer transferring. Omitting the ion implantation and epitaxial growth processes facilitates the manufacturing of silicon thin-film solar cells, thick SOI wafers, and MEMS components by making this method highly cost-efficient.
